# Mannan Oligosaccharides Promoted Skeletal Muscle Hypertrophy through the Gut Microbiome and Microbial Metabolites in Mice

**DOI:** 10.3390/foods12020357

**Published:** 2023-01-12

**Authors:** Weijie Zhao, Lvshuang Chen, Weihao Tan, Yongxiang Li, Lijuan Sun, Xiaotong Zhu, Songbo Wang, Ping Gao, Canjun Zhu, Gang Shu, Lina Wang, Qingyan Jiang

**Affiliations:** 1 Guangdong Provincial Key Laboratory of Animal Nutrition Control, College of Animal Science, South China Agricultural University, Guangzhou 510642, China; 2 National Engineering Research Center for Breeding Swine Industry, South China Agricultural University, Guangzhou 510642, China

**Keywords:** C2C12, decanoic acid, mannan oligosaccharides, metabolite profile, skeletal muscle, gut microbiome

## Abstract

Mannan oligosaccharides (MOSs) have been implicated in the animal growth rate, health indices, and lipid oxidative stability. MOSs have been indicated to maintain intestinal health and anti-inflammatory effects via modulation of gut microbiota. Furthermore, the role of MOSs in modulating skeletal muscle function is largely unknown. Here, this study aimed to investigate the effects of MOS supplementation on muscle function and muscle mass in mice. Additionally, the possible underlying mechanisms, including the contributions of gut microbiota and microbial metabolites, were explored. In our study, 3-week-old C57BL/6J male mice (body weight of approximately 10.7 ± 1.1 g) were given pure water or pure water with 1% MOS. To study the effect of MOSs on gut-microbiota-derived metabolites, serum metabolic profiles were analyzed through untargeted metabolomic profiling. Moreover, we detected the downstream signals of differential metabolites, and decanoic acid (DA) was selected as our target spot. Then, DA was used to treat C2C12 cells, and we found that DA promotes C2C12 cell differentiation via the GPR84 and PI3K/AKT signaling pathways. In conclusion, these results showed that MOS supplementation improves muscle function and muscle mass. Additionally, gut microbiome and microbial metabolites were regulated by MOSs, and DA may be one of the most important links between the gut microbiome and skeletal muscle function regulation.

## 1. Introduction

Muscle development directly influences growth and health in humans and animals [1,2]. Therefore, altering the capacity and/or efficiency of muscle growth is very important [3]. Skeletal muscle is a highly adaptive tissue with plastic properties. Skeletal muscle is capable of altering its phenotype in response to external stimuli, including physiological stimuli, atrophy, disease, exercise and injury [4]. Exercise, hormonal and nutritional levels may all be involved in skeletal muscle hypertrophy and increase skeletal muscle mass. A variety of signaling molecules are involved in skeletal muscle hypertrophy. Insulin-like growth factor-1 (IGF-1) increases skeletal muscle hypertrophy via the PI3K/Akt/mTOR and PI3K/Akt/GSK3β pathways [5]. Recently, transplantation of the gut microbiota from pathogen-free mice into germ-free mice resulted in an improvement in germ-free mouse skeletal muscle atrophy [6]. This shows that the gut microbiome may influence the physiological functions of muscles.

Mannan oligosaccharides (MOSs) are low-molecular-weight carbohydrates, and the degree of polymerization can vary from 2 to 10 [7]. MOSs are nondigestible carbohydrates that cannot be hydrolyzed by pancreatic amylases but can be degraded by enzymes produced by the gut microbiome [8]. Thus, MOSs have no direct nutritive value, but they have been shown to be able to have a positive effect on the performance of animals [9]. MOSs are being utilized for the modulation of gut microbiota, and microbial metabolites, such as bile acids (BAs) and short-chain fatty acids (SCFAs), play an important role in improving human and animal health [10]. Studies of other carbohydrates have found similar results. Epilactose (a rare nondigestible disaccharide) can increase in UCP-1 in the skeletal muscle through propionic acid (a bacterial metabolite) [11]. Chitosan oligosaccharides promoted blood perfusion and neovascularization in the ischemic hindlimb muscle of mice. The changed gut microbiome and microbial metabolites might play an important role in this process [12]. MOSs have been indicated to maintain intestinal health and anti-inflammatory activity [13], while there is little research on the effect of MOSs on skeletal muscle function.

Thus, this study aimed to investigate the effects of MOS supplementation on muscle function and muscle mass in mice. In addition, the possible underlying mechanisms, including the contributions of gut microbiota and microbial metabolites, were explored. Furthermore, the selected metabolites were verified in C2C12 cells. Our data showed that MOS supplementation may activate the GPR84 and PI3K/AKT signaling pathways in skeletal muscle by increasing the level of DA in serum, thereby improving muscle fiber types and muscle function.

## 2. Materials and Methods

### 2.1. Animals and Experimental Design

All animal experiments were conducted with the permission number SYXK (Guangdong) 2019-0136.

Twelve 3-week-old C57BL/6J male mice (body weight 10.7 ± 1.1 g) were purchased from Guangdong Medical Laboratory Animal Center. The mice were housed in environmentally controlled rooms on a 12 h light–dark cycle.

The body weight of the mice was measured weekly. The experiment lasted 8 weeks. At the end of treatment, the mice were sacrificed by carbon dioxide anesthesia. The serum was collected and stored at −20 °C. Meanwhile, the gastrocnemius (GAS), soleus (Sol), tibialis anterior (TA) and extensor digitorum longus (EDL) were collected and weighed. These samples were stored at −80 °C until further analyses.

### 2.2. Dosage Information

The mice were randomly divided into two groups (*n* = 6). There was free feeding (diet composition in Table 1) and drinking of mice. The control group was given pure water. The MOS group was given pure water with 1% MOS (*w*/*w*). The MOS (the purity is 96.1%, 61.3% have a polymerization degree of 2–6, and the rest have a polymerization degree of 7–10) was purchased from Shaanxi Scipher Natural Products Co., Ltd. (Xi’an, China).

### 2.3. Body Composition

After 7 weeks of treatment, the body composition was assessed by using an NMR Analyzer (MesoQMR23-060H, Niumag Corporation, Shanghai, China).

### 2.4. Grip Strength and Weight Test

After the mice were fed for 7 weeks, the grip strength test was performed as previously described [14]. After the grip strength test, the mice rested for 3 days. Weight tests were performed as described previously [6].

### 2.5. Staining of ATPase

Staining of ATPase in GAS and cell count were performed as previously described [15].

### 2.6. Serum Metabolite Profile Assessment

Serum metabolic profiles were analyzed by untargeted metabolomic profiling using UPLC–MS/MS at Metaboprofile Biotechnology Co., Ltd. (Shanghai, China) [16].

### 2.7. 16S rRNA Microbiome Analysis

After 7 weeks of treatment, feces were collected in sterile centrifuge tubes and stored at −80° for further analyses. The fecal microbiome was sequenced by Metaboprofile Biotechnology Co., Ltd. (Shanghai, China). Fecal sample preparation and the sequencing protocol were based on a previously published method [17].

### 2.8. Western Blot Analysis

Western blotting was conducted as previously described [18]. A total of 20 μg of protein was loaded. The primary antibodies used included FXR (ab235094, Abcam PLC, Cambridge, UK), TGR5 (NBP2-23669, Novus Biologicals, Littleton, CO, USA), GPR84 (bs-13507R, Bioss, Beijing, China), TLR4 (sc-293072, Santa Cruz Biotechnology, Santa Cruz, CA, USA), p-AKT/AKT (#4060/9272s, CST, Danvers, MA, USA) and p-PI3K/PI3K (310163/R22768, ZEN-BIOSCIENCE, Chengdu, China).

### 2.9. Enzyme-Linked Immunosorbent Assay (ELISA)

cAMP and L-carnosine were detected by using ELISA kits according to the manufacturer’s instructions (Shanghai Ruifan Biotechnology Co., Ltd., Shanghai, China). The detection limits of the cAMP ELISA kit were 0–12 nmol/mL. The detection limits of the L-carnosine ELISA kit were 1–48 pmol/L.

### 2.10. Cell Culture and Treatment

The mouse myoblast cell line C2C12 was cultured based on a previously published method [15]. When cells reached 50% confluency, they were transfected with GPR84 siRNA (Guangzhou RiboBio Co., Ltd., Guangzhou, China). The transfection procedure was carried out according to the instructions of the manufacturer. When the cells reached 80% confluency, the culture media was switched to high glucose DMEM with 2% horse serum and 100 μM DA to induce myoblast differentiation into myotubes for 6 days. After treatment, the cells were harvested for further analyses.

### 2.11. Immunocytochemistry

The immunocytochemistry of C2C12 cells was performed as previously described [19]. The primary antibody used was MyHC (MAB4470, R&D Systems, Minneapolis, MN, USA). The secondary antibody used was goat anti-mouse IgM/Alexa Fluor 555 antibody (bs-0368G-AF555, Bioss, Beijing, China). Images were captured with a microscope at 20 X magnification (6 fields per sample captured), and the myotube diameter and myotube fusion index were counted by using IPP.

### 2.12. Statistical Analysis

Data are expressed as the mean ± S.E.M. Significance comparisons were performed using Student’s *t*-test and one-way ANOVA in Graphpad Prism 8.0, with *p* < 0.05 indicating a significant difference.

## 3. Results

### 3.1. Mannan Oligosaccharide Supplementation Shows Positive Effects on the Gastrocnemius Muscle

**Figure 1:**
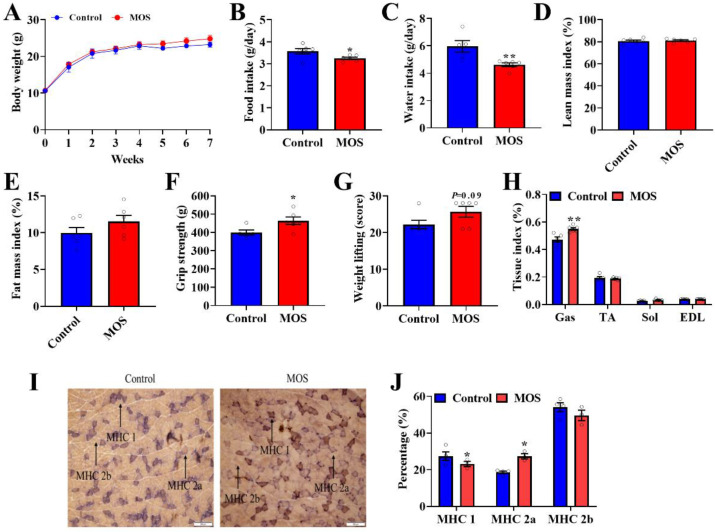
Mannan oligosaccharide supplementation shows positive effects on the gastrocnemius. (**A**) Body weight of mice. (**B**) Average daily food intake. (**C**) Average daily water intake. (**D**,**E**) QMR analyses of body composition of mice. (**F**) The grip strength of mice. (**G**) The weight test of mice. (**H**) The GAS, TA, SOL and EDL tissue indices of mice. (**I**,**J**) Staining of ATPase in GAS. * *p* < 0.05 versus the control group, ** *p* < 0.01 versus the control group.

First of all, the role of MOS supplementation on growth and body composition in mice was investigated. Although MOS supplementation significantly decreased food intake and water intake (Figure 1B,C), it had no effect on body weight or body composition in mice (Figure 1A,D,E). Through the grip strength test and weight lifting test on mice, we found that the grip strength and weight lifting of mice were significantly improved in the MOS group (Figure 1F,G). Moreover, the GAS index was increased in the MOS group (Figure 1H). To explore the possible reasons for the enhancement of muscle function, the gastrocnemius was histologically stained. Staining of ATPase in GAS showed that the proportion of MHC 1 expression was decreased, while MHC 2a expression was increased in the MOS group (Figure 1I,J).

### 3.2. Serum Metabolic Profiles of Mice Fed Mannan Oligosaccharides

**Figure 2:**
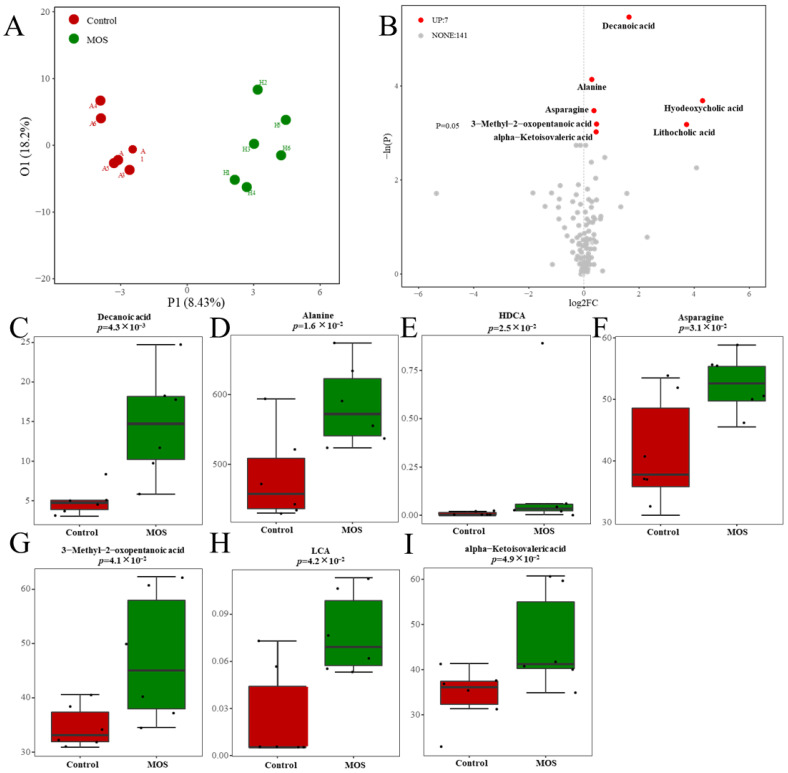
Serum metabolic profiles of mice fed mannan oligosaccharides. (**A**) The serum metabolome by OPLS–DA. (**B**) Metabolites in mice serum of volcano plot. (**C**–**I**) The box charts show the detailed serum metabolites and significant changes between the control and MOS groups (μM). Up, upregulated; FC, fold change.

According to previous studies, MOSs resist the hydrolysis of intestinal digestive enzymes and regulate organism metabolism through the decomposition of the gut microbiome [20]. To study the effect of MOSs on gut-microbiota-derived metabolites, serum metabolic profiles were analyzed through untargeted metabolomic profiling by using a UPLC–MS/MS system. The differential metabolites were analyzed by orthogonal partial least squares discriminant analysis (OPLS–DA). The results showed that there was a distinct difference between the control and MOS groups (Figure 2A), which means that MOS supplementation influenced the serum metabolic profile in mice. As shown in Figure 2B, 148 gut microbiome metabolites were detected in the control and MOS groups. Among these metabolites, seven gut microbiome metabolites were significantly upregulated in the MOS group. Specifically, gut microbiome metabolites including decanoic acid (DA, FC value = 1.63), alanine (FC value = 1.21), hyodeoxycholic acid (HDCA, FC value = 19.67), asparagine (FC value = 1.27), lithocholic acid (LCA, FC value = 13.12), 3-methyl-2-oxopentanoic acid (FC value = 1.36) and alpha-ketoisovaleric acid (FC value = 1.35) were significantly upregulated (Figure 2C–I).

### 3.3. The Gut Microbiome of Mice Fed Mannan Oligosaccharides

**Figure 3:**
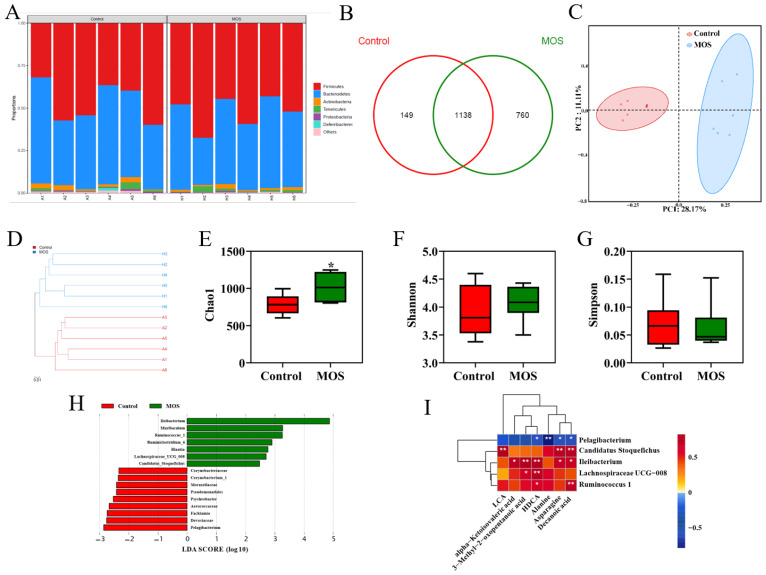
Gut microbiome of mice fed mannan oligosaccharides. (**A**) Relative abundance analysis at the phylum level. (**B**) OTU Venn analysis. (**C**) Principal coordinate analysis (PCOA). (**D**) Hierarchical clustering analysis. (**E**) Chao1 value. (**F**,**G**) Shannon and Simpson index of gut microbiota. (**H**) LDA score of gut microbiota. (**I**) Correlations between serum differential metabolites and differential gut microbiota. * *p* < 0.05 versus the control group.

To investigate the effects of MOS supplementation on the gut microbiome of mice, the fecal microbiota composition was evaluated by 16S rRNA gene sequencing. The relative abundance analysis at the phylum level showed no apparent differences (Figure 3A). OTU Venn analysis, principal coordinate analysis and hierarchical clustering analysis showed differences in the microbiome (Figure 3B–D). As shown in Figure 3E, the MOS group had higher community richness. However, MOS supplementation had no significant differences in microbiota community diversity (Figure 3F,G). LEfSe was used for linear discriminant analysis (LDA) to identify significant differences in the abundance of the microbiota. The different species (LDA score > 2, *p* < 0.05) are shown in Figure 3H. Spearman correlation analysis was conducted between the serum differential metabolites (Figure 2A) and the differential microbiome (Figure 3D). Interestingly, there was a positive correlation between the abundances of specific microbiota (*Candidatus_Stoquefichus, Ileibacterium, Lachnospiraceae_UCG_008 and Ruminococcus_1*), which were significantly increased, and metabolites were significantly increased in the MOS group (Figure 3I). In contrast, there was a negative correlation between the abundance of the microbiota (*Pelagibacterium*), which was increased significantly in the control group, and specific metabolites (DA, asparagine, alanine and HDCA) (Figure 3I). These results indicated that these differential microbiomes were closely associated with and might contribute to the altered serum metabolic profiles in response to MOS supplementation.

### 3.4. Decanoic Acid May Be the Key Metabolite That Mediates the Effects of Mannan Oligosaccharides on Skeletal Muscle

**Figure 4:**
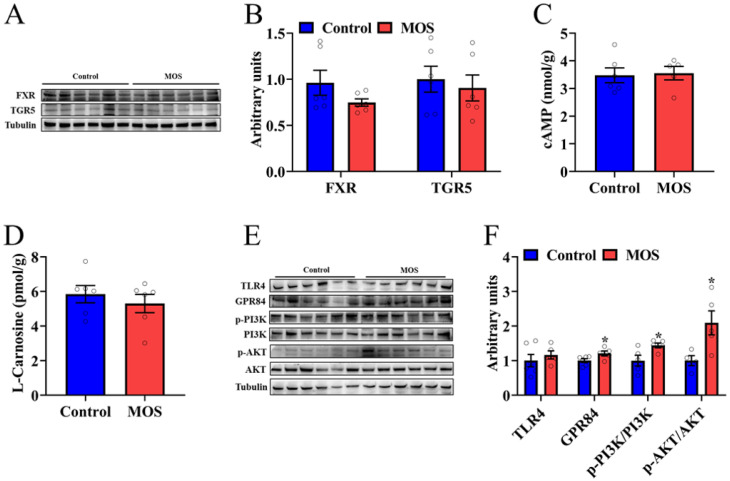
Decanoic acid may be the key metabolite that mediates the effects of mannan oligosaccharides on skeletal muscle. (**A**,**B**) The protein expression level of FXR and TGR5 in the GAS. (**C**,**D**) cAMP and L-carnosine concentrations in the GAS. (**E**,**F**) The protein expression level of TLR4, GPR84 and PI3K/AKT signaling pathways in the GAS. * *p* < 0.05 versus the control group.

To explore which metabolite caused an increase in mouse skeletal muscle function, we examined the downstream signals of differential metabolites. Among the seven significantly changed metabolites, asparagine, 3-methyl-2-oxopentanoic acid and alpha-ketoisovaleric acid had a lower fold change than the other metabolites, so we will focus on DA, alanine, HDCA and LCA. HDCA and LCA, as secondary bile acids, play a role in organism metabolism through bile acid receptors. Therefore, the protein expression levels of FXR and TGR in the GAS were detected. Western blot analysis revealed that the expression of FXR and TGR5 was not significantly different (Figure 4A,B). In addition, the cAMP level in the gastrocnemius was not significantly different, which is a downstream signaling molecule of TGR5 (Figure 4C). There was no significant difference in the L-carnosine level, which is composed of alanine and histidine (Figure 4D). The level of expression of MCFA receptors was detected, and GPR84 was significantly increased in the MOS group (Figure 4E,F). Furthermore, the PI3K/AKT signaling pathway was activated in the MOS group (Figure 4E,F). In summary, we speculate that MOS supplementation may activate the GPR84 and PI3K/AKT signaling pathways in GAS by increasing the level of DA in serum.

### 3.5. Decanoic Acid Mediates C2C12 Cell Differentiation through the GPR84 and PI3K/AKT Signaling Pathways

**Figure 5:**
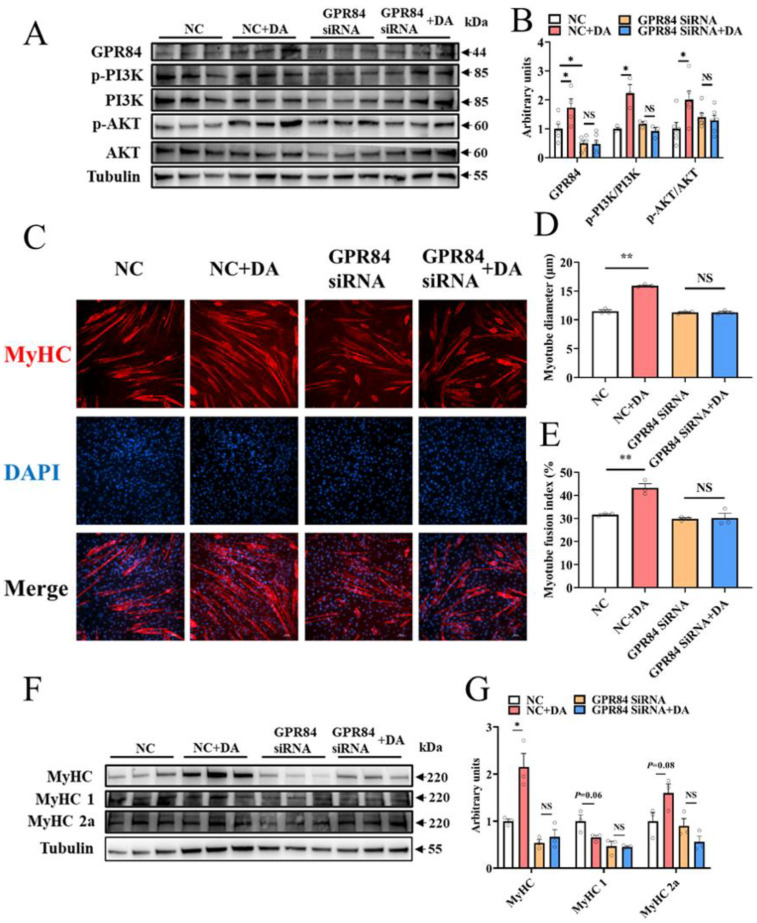
Decanoic acid mediates C2C12 cell differentiation through the GPR84 and PI3K/AKT signaling pathways. (**A**,**B**) The protein expression level of GPR84 and PI3K/AKT signaling pathways in C2C12 cells. (**C**–**E**) Immunocytochemistry of myotubes in C2C12 cells. Red: MyHC; blue: DAPI. (**F**,**G**) The protein expression level of MyHC, MyHC 1 and MyHC 2a in C2C12 cells. NS indicates no significant differences. * *p* < 0.05 versus the control group, ** *p* < 0.01 versus the control group.

To prove the regulatory effect of DA on skeletal muscle, DA was used to treat C2C12 cells, and the results were consistent with those in mice. After DA treatment, the protein expression level of GPR84 was significantly upregulated, and the PI3K/AKT signaling pathway was activated (Figure 5A,B). Additionally, immunocytochemistry of C2C12 cells showed that DA significantly increased the myotube diameter and myotube fusion index (Figure 5C–E). Moreover, the protein expression of MyHC was increased significantly (Figure 5F,G), and MyHC 1 had a tendency to convert to MyHC 2a (Figure 5F,G). To further confirm that GPR84 plays a role in DA-mediated promotion of C2C12 cell differentiation, GPR84 siRNA was used to knockdown the expression of GPR84. As expected, the protein expression of GPR84 was significantly decreased by GPR84 siRNA, while the phosphorylation levels of PI3K and AKT were not significantly different (Figure 5A,B). Consistently, the increase in C2C12 cell differentiation induced by DA was eliminated by GPR84 siRNA (Figure 5C–G). These results demonstrated that DA promotes C2C12 cell differentiation via GPR84.

## 4. Discussion

Consistent with previous reports, we found that MOS supplementation had no discernible effect on body weight or composition [21]. Significantly better body weight gain and feed conversion ratios were observed in broiler chickens supplemented with MOS [22]. In our study, the decrease in food intake and water intake also proves that MOSs decrease the feed conversion ratio. This may be due to MOSs improving nutrient availability and absorption. A previous study has shown that type 2 fibers have the largest diameter and highest myofibrillar and ATPase activities [23]. In this study, MOS supplementation decreased the proportion of type I muscle fibers while increasing the proportion of type IIa muscle fibers in the GAS. Therefore, we speculate that the increase in muscle function and mass is due to the increase in the proportion of IIa muscle fibers. The synbiotic-supplemented (comprising MOSs) diet alters the fatty acid composition of broiler muscles [24]. Dietary supplementation with chitosan and galacto-mannan-oligosaccharides may improve growth and feed conversion by increasing plasma IGF-I levels and increasing muscle IGF-1 mRNA expression levels in piglets [25]. In this study, supplemented MOSs increased MCFA (DA) levels in serum. This study found that supplemented MOSs activate the AKT/PI3K signaling pathway downstream of IGF-1.

Because MOS supplementation affects muscle function and muscle mass, we further explored the possible underlying mechanisms. One study indicates that MOSs can help the proliferation of beneficial microbes to improve animal health [26]. Our results showed that the MOS group had more microbiota species. MOSs could improve health by modulating the composition of the gut microbiome and by changing microbial metabolites such as SCFAs in the feces and serum [27,28]. Although MOS supplementation reshaped the gut microbiota in mice, the composition of the gut microbiota and microbial metabolites were different from those in these previous studies, which might be explained by the differences in the dietary pattern, the composition of treatment, the experiment duration and the feeding environment.

In the gut, the microbiota can produce many substances, such as fatty acids and bile acids, which contribute to the physiology of the host [29]. These metabolites exert their effects within the host as signaling molecules and substrates for metabolic reactions [30]. In myostatin-edited Large White pigs, there was a significant correlation between *Candidatus_Stoquefichus* and glycerophospholipid metabolites [31]. It has been reported that in LPS-challenged piglets, LPS disturbs the normal enterohepatic circulation of bile acids, which may be due to LPS changing operational taxonomic units related to *Ileibacterium* [32]. *Lachnospiraceae UCG-008* can enhance the utilization efficiencies of cellulose and hemicellulose from native forage [33]. Other studies have shown that serum bile acid levels were significantly correlated with the abundance of *Ruminococcus_1* (T. Li et al., 2021). This is similar to our research, in which these bacteria were correlated with changes in metabolites.

In our study, a total of seven microbial metabolites were changed, including DA, alanine, HDCA, LCA, asparagine, 3-methyl-2-oxopentanoic acid and alpha-ketoisovaleric acid. These latter three metabolites are intermediate products of amino acid metabolism, and there are fewer reports related to skeletal muscle [34,35,36]. In our study, these metabolites had a lower fold change and *p* value than other metabolites, so we focused on other metabolites. HDCA and LCA, as secondary bile acids, play a role in organism metabolism through bile acid receptors [37]. Other studies have shown that the BA receptors FXR and TGR5 can regulate skeletal muscle function and metabolism [38]. Alanine combines with histidine to form L-carnosine, which relieves muscle damage caused by oxidative stress [39]. DA increases the capacity for fatty acid oxidation in skeletal muscle [40]. Although these metabolites changed, only the downstream signals of DA changed accordingly. We speculate that although the changes in these metabolites are significantly different, they are still not enough to cause changes in downstream signals. DA had the largest change, with an average increase of approximately three times, and thus caused a change in the downstream signal.

GPR84 has high expression in skeletal muscle as an MCFA receptor and plays an important role in regulating mitochondrial function [41]. It has been demonstrated that lauric-acid-induced activation of the PI3K/AKT signaling pathway was inhibited by GPR84 siRNA [42]. Consistent with previous reports, DA-induced activation of the PI3K/AKT signaling pathway was inhibited by GPR84 siRNA. It has been reported that activation of the PI3K/AKT signaling pathway promotes skeletal muscle hypertrophy and mass [43]. In this study, MOS supplementation enhanced muscle function and muscle mass in mice and activated the PI3K/AKT signaling pathway. Similar results were found in cell experiments, in which DA treatment promoted the differentiation of C2C12 cells by activating the PI3K/AKT signaling pathway.

## 5. Conclusions

In conclusion, MOS supplementation improves muscle function and muscle mass. Additionally, gut microbiome and microbial metabolites were regulated by MOSs, and DA may be one of the most important links between the gut microbiome and skeletal muscle function regulation. Specifically, we speculate that MOS supplementation may activate the GPR84 and PI3K/AKT signaling pathways in skeletal muscle by increasing the level of DA in serum, thereby improving muscle mass and muscle function.

## Figures and Tables

**Table 1:** Diet composition (g/kg diet).

Ingredient	Content
Casein	140.0
L-cystine	1.8
Maltodextrin	155.0
Sucrose	100
Cellulose	50.0
Mineral mix	35.0
Vitamin mix	10.0
Choline chloride	2.5
Soy oil	40.0
Corn starch	465.7
Gross energy (kcal/g)	3.60

## Data Availability

Data available on request due to restrictions eg privacy or ethical.
